# Posterior Reversible Encephalopathy Syndrome After Self-Medication With an Oral Decongestant: A Case Report

**DOI:** 10.3389/fmed.2022.837324

**Published:** 2022-03-07

**Authors:** Yoann Zerbib, Louis Gibert, Youssef Bennis, Kamel Masmoudi, Julien Maizel, Clément Brault

**Affiliations:** ^1^Intensive Care Unit, Amiens University Medical Center, Amiens, France; ^2^Department of Clinical Pharmacology, Amiens University Medical Center, Amiens, France; ^3^MP3CV Laboratory, UR UPJV 7517, Amiens, France

**Keywords:** posterior reversible encephalopathy syndrome, oral decongestant, self-medication, end-stage renal disease, sepsis

## Abstract

**Introduction:**

Posterior reversible encephalopathy syndrome (PRES) is a rare neurological disorder caused by the dysregulation of cerebral perfusion.

**Case Presentation:**

We report on a 18-year-old female patient with a history of end-stage renal disease and thrice weekly hemodialysis. She was admitted to the emergency department with mental confusion, blurred vision, headaches, and vomiting, following self-medication with an oral decongestant containing pseudoephedrine. We observed hypointense lesions with T1-weighted MRI and hyperintense areas with T2-weighted and fluid-attenuated inversion recovery MRI sequences. The lack of diffusion restriction was consistent with a diagnosis of PRES. A concomitant *Enterobacter cloacae* hemodialysis catheter-bloodstream infection was also diagnosed. We hypothesize that both sepsis and inappropriate self-medication with oral pseudoephedrine contributed to hypertension, endothelial dysfunction, and vasogenic edema. The patient received intensive care and made a full recovery.

**Discussion:**

PRES is a life-threatening condition that requires intensive care. Identification of the etiology is the keystone of medical care. Inappropriate self-medication with an oral decongestant might trigger PRES - highlighting the importance of patient education.

## Introduction

Posterior reversible encephalopathy syndrome (PRES) is a rare neurological disorder caused by the dysregulation of cerebral perfusion (1). The main clinical features include headaches, vision impairment, altered consciousness, and seizures (2). The most common radiological finding is reversible vasogenic edema in the posterior cerebral circulation; this is visible as hypodense lesions in non-contrast-enhanced computed tomography, hypointense lesions in T1-weighted MRI, and hyperintense areas in T2-weighted and fluid-attenuated inversion recovery (FLAIR) MRI, with the absence of diffusion restriction (3).

Here, we report on a patient who developed PRES after self-medication with an oral decongestant. This case emphasizes the need to (i) promptly recognize the etiology and (ii) prevent inappropriate self-medication.

## Case Report

### Clinical Findings

An 18-year-old female was admitted to our emergency department with mental confusion, blurred vision, headaches, and vomiting. Congenital kidney hypoplasia had been discovered late and had resulted in end-stage renal disease (ESRD) with anuria, thrice-weekly hemodialysis, and arterial hypertension [controlled with bisoprolol (5 mg/d) and urapidil (120 mg/d)]. On the day before admission, the patient had self-medicated with a dose of oral decongestant containing 60 mg pseudoephedrine (PDE) and 500 mg acetaminophen.

The patient's vital data on admission were as follows: systolic/diastolic blood pressure: 210/100 mmHg: body temperature: 37.1°C; body weight: 48 kg; height: 1.63 m; capillary blood glucose: 0.9 g/L. The patient's cardiac and respiratory signs were unremarkable. We noted the presence of purulent discharge at the hemodialysis catheter's insertion site. A neurological examination showed bilateral vision loss and altered mental status, with agitation and delirium. No limb weakness or irritative pyramidal signs were seen. Blood tests showed normal levels of sodium, potassium and magnesium. As expected, the serum creatinine level was elevated (768 μmol/L). We observed type B lactic acidosis, with an elevated blood lactate level (8 mmol/L; normal range: 0.5–2 mmol/L), a low pH (7.24; normal range: 7.38–7.42), and a low serum bicarbonate level (18 mmol/L; normal range: 24–32 mmoL/L). The serum C-reactive protein (CRP) level was slightly elevated, and the liver function tests were normal. A blood culture seeded with a sample taken from the hemodialysis catheter at admission was positive for *Enterobacter cloacae*.

To complete further examinations, the patient had to be sedated and ventilated. An analysis of the cerebrospinal fluid (CSF) did not evidence signs of infection, inflammation or pleocytosis. The CSF protein and glucose levels were within the normal range.

**Figure 1:**
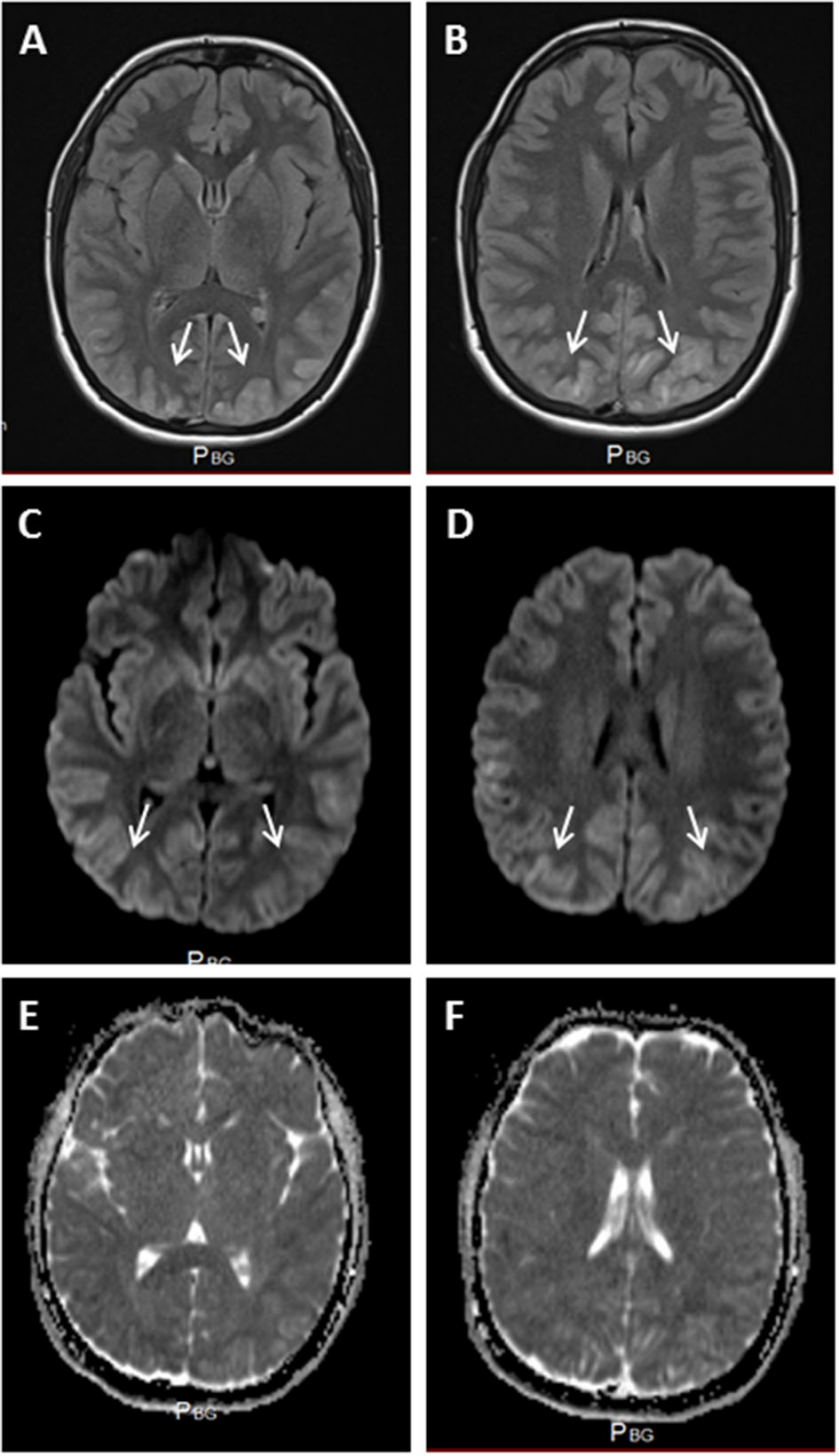
**(A,B)** FLAIR MRI, showing bilateral hyperintensities in the occipital lobes, with cortical edema (white arrows). Hyperintense areas were also apparent in diffusion-weighted sequences **(C,D)** (white arrows), with no apparent diffusion restriction **(E,F)**.

Although the brain computed tomography results were normal, T2, FLAIR and diffusion cerebral MRI sequences evidenced bilateral hyperintense occipitotemporoparietal lesions and thickening of the cortex. These observations were consistent with vasogenic edema (Figure 1).

### Toxicological Analysis

**Figure 2:**
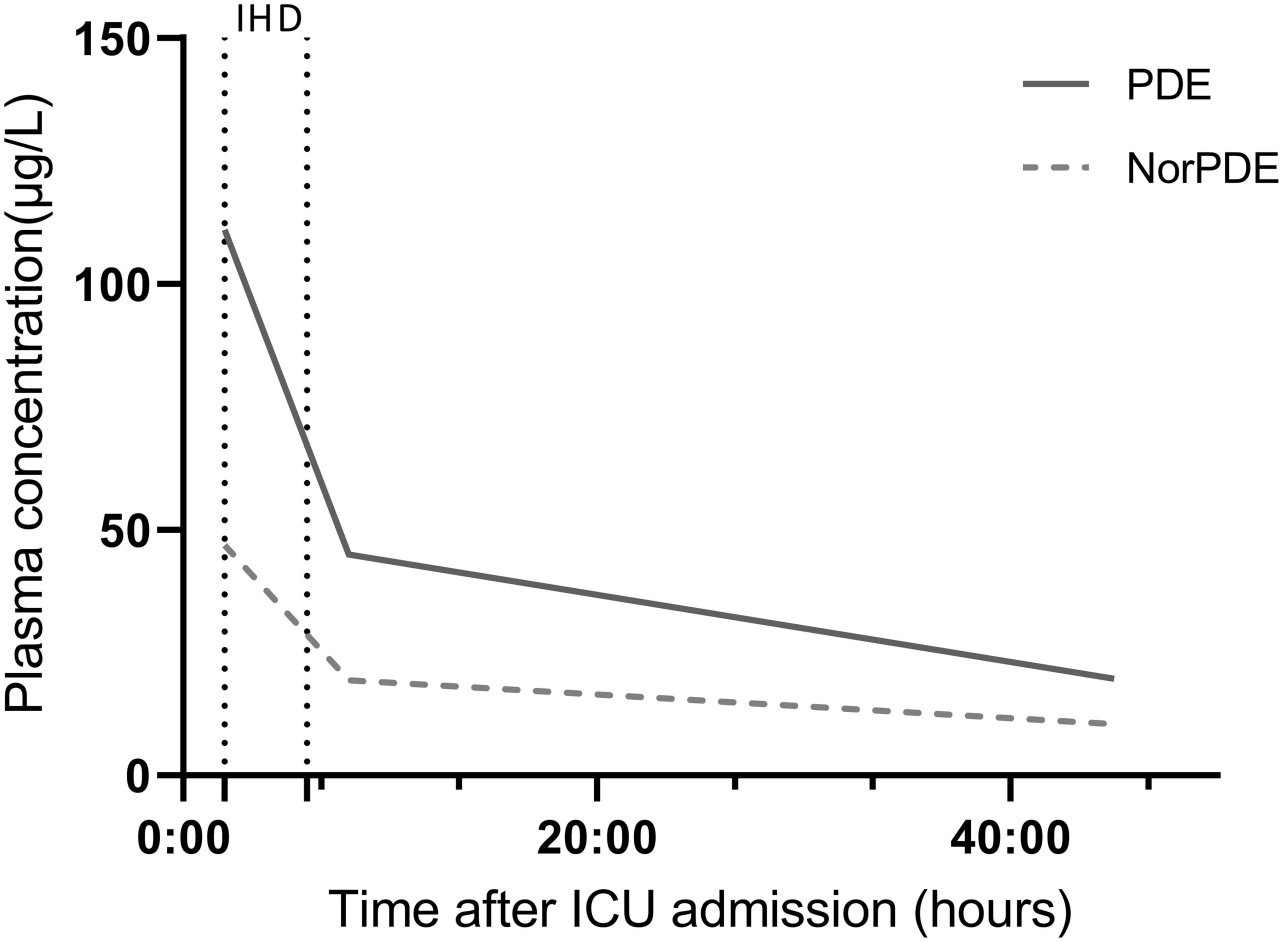
Changes over time in plasma PDE and norPDE concentrations, as measured using high-performance liquid chromatography coupled with tandem mass spectrometry. IHD, intermittent hemodialysis.

A toxicological analysis (high-performance liquid chromatography coupled with tandem mass spectrometry) on admission detected acetaminophen, PDE and norpseudoephedrine (norPDE). The plasma concentrations of acetaminophen, PDE and norPDE were, respectively 4.4 mg/L and 111 and 46.8 μg/L (Figure 2).

### Therapeutic Interventions and Follow-Up

After admission to the intensive care unit (ICU), the patient was given a continuous intravenous infusion of nicardipine for close blood pressure control. Renal replacement therapy (intermittent hemodialysis; 4 h, 3 times a week) was initiated on admission. The lactic acidosis had resolved 2 h post-admission. The plasma PDE and norPDE levels fell, respectively to 44.9 and 19.3 μg/L after the first intermittent hemodialysis session and to then 19.7 and 10.4 μg/L 37 h later. Assuming single-phase exponential decay of the plasma concentration, we estimated that the elimination half-lives of PDE and norPDE were normal during hemodialysis (6.1 and 6.3 h for PDE and norPDE, respectively, compared with 5–8 h in healthy volunteers) and greatly lengthened in the absence of hemodialysis (31.1 and 41.5 h, respectively) (4).

Continuous piperacillin-tazobactam infusion was also initiated, with a switch to piperacillin alone for treatment of the *Enterobacter cloacae* bloodstream infection. The hemodialysis catheter was replaced 4 h after ICU admission, and the hemodialysis sessions were continued. Neurological symptoms started to improve from day 2 and the patient was discharged from the ICU 6 days after admission to hospital. After 6 days of follow-up after ICU discharge, the patient's blood pressure was under control and the results of a neurological examination were unremarkable. MRI did not evidence any signal abnormalities and showed that the vasogenic edema had completely regressed.

## Discussion

Here, we reported on a case of PRES associated with a catheter bloodstream infection and self-medication with PDE in a patient with ESRD and hypertension. The pathophysiology of PRES is complex and has not been fully elucidated. It is acknowledged that elevated intracranial pressure and endothelial injury can disrupt the blood-brain barrier (5–7). In the present case, uncontrolled hypertension from oral decongestant in the setting of renal failure and sepsis might have triggered PRES.

In France and elsewhere, PDE is commonly available in over-the-counter oral decongestants. The molecule is fully absorbed from the gastrointestinal tract. The plasma PDE concentration peaks around 2 h after oral administration (180 μg/l on average, after a 60 mg dose) (8). Most of the PDE is eliminated by the kidneys. Acute or chronic renal failure therefore decreases plasma clearance of PDE and prolongs the drug's half-life *in vivo* (4). On the basis of our observations, we suspect that the greatly extended half-life and the concomitant sepsis may have trigger PRES in this patient with chronic hypertension and ESRD.

There are few published data on the relationship between the plasma or serum concentrations of PDE and blood pressure. The PDE peak appeared to be associated with a significant increase in systolic blood pressure (9). One can reasonably assume that the extension of PDE's half-life - even at normal therapeutic doses - would cause regional vasoconstriction and an increase in intracranial blood pressure. Indeed, several accidental overdoses of PDE have been described, with a hypertensive crisis, coronary vasospasm, acute myocardial infarction, or acute ischemic colitis (10, 11). Only one report mention an association between PDE administration and PRES (12).

There is growing body of evidence to suggest that the administration of oral decongestants can have life-threatening complications. Nevertheless, these drugs are available over the counter in many countries. Over-the-counter medications are usually considered to be safe and effective, and self-care is often judged to be an essential part of any healthcare system. However, clinicians and pharmacists should be aware of the potential complications and should educate patients with major risk factors, such as ESRD.

## Conclusion

PRES is a life-threatening condition that requires intensive care. We hypothesize that both sepsis and inappropriate self-medication with an oral decongestant contributed to hypertension, endothelial dysfunction and vasogenic edema in the present case.

## Data Availability Statement

The original contributions presented in the study are included in the article/supplementary material, further inquiries can be directed to the corresponding authors.

## Ethics Statement

Written informed consent was obtained from the individual(s) for the publication of any potentially identifiable images or data included in this article.

## Author Contributions

YZ, LG, and YB drafted the manuscript. KM, JM, and CB revised the manuscript for critical intellectual content. All the authors approved the final version of the manuscript.

## Conflict of Interest

The authors declare that the research was conducted in the absence of any commercial or financial relationships that could be construed as a potential conflict of interest.

## Publisher's Note

All claims expressed in this article are solely those of the authors and do not necessarily represent those of their affiliated organizations, or those of the publisher, the editors and the reviewers. Any product that may be evaluated in this article, or claim that may be made by its manufacturer, is not guaranteed or endorsed by the publisher.
